# Psychiatry

**Published:** 1905-09-30

**Authors:** 


### PSYCHIATRY.

**Hallucinations in Insanity.**—The generally accepted distinction made by Esquirol a century ago between illusions and hallucinations is now known not to be correct. Esquirol believed that a hallucination had its seat in and arose as it were spontaneously from one or other of the "psycho-sensorial" centres of the brain, and that this happened in the absence of any objective stimulus. Recently Dr. W. A. White, of New York,[1] has pointed out that this and many other views current since the time of Esquirol have been found inadequate to explain hallucinations. The view advanced by White corresponds to that known as the theory of "photisms and phonisms," the facts and explanation of which are now well established. The theory of Dr. White asserts that hallucinations do not arise primarily and *de novo* in the higher sensorial centres which constitute part of the ego, but are secondarily excited therein by stimuli arising from without, *i.e.* from other afferent sensorial tracts, and that hallucinations are of the nature of "photisms," which may be called up by stimuli of any other senses (*e.g.* taste, hearing, tactile stimuli), or of phonisms similarly aroused by various other sensorial excitations than sound. This theory, says Dr. White, was first propounded by Dr. Boris Sidis, of Baltimore, and "holds within it the explanation of hallucinations." The author presents in considerable detail ten cases bearing out his views and showing how the phenomena (of photisms, phonism, and analogous processes) as ordinarily experienced "can graduate into a true hallucination." A study of these cases has led him to the conclusion, already reached in other ways by many recent students and investigators of the same subject, that hallucinations are higher cortical or psychical events following excitations of one or more lower afferent paths leading up to the centre, which is thus roused into hallucinosis. The mental state, therefore, is logically identical in hallucinations and illusions, for, given the sensory-afferent elements, the falseness of their interpretation or apperception is due to central (psychico-cortical) derangement. Two clinical points are emphasised. Many sensations, either because of their weakness or because of the preponderance of some other sensation *pro tem.*, may not become appreciated; and, again, sensations that under ordinary circumstances would hardly reach the threshold of consciousness may, from some reason or other—from the occasional or periodic upheaval of passion and organic cravings of the body—acquire unusual power, and thus rising into prepotency become for the time a hallucination or an irresistible obsession. This occurs at times in some of the "borderland" cases of obsessional insanity in degenerates, and may even occur in normal cases of disturbance of the dream consciousness. It is necessary in order that the particular hallucination be developed to perfection that there should be a certain anticipatory "preparedness" on the part of the mind, and this is not more clearly shown than in the systematisation of hallucinations into logical schemes of delusion in the mind of the paranoiac. A patient affected with tinnitus aurium has his mind attuned to, and is prone to facility of development of, auditory hallucinations. In conclusion, Dr. White says he has never failed to find a peripheral exciting cause in all hallucinated cases he has examined. Such causes explained indirectly or directly the phenomena of the hallucination, and he would be averse to other than a "peripheral" origin of the cause in a hallucinated case.

**Visual Disorders in Insanity.**—Dr. Sydney Cole [2] deals with the subject of disorders of visual association in insanity. The normal individual, he says, orientates himself in his environment mainly by the help of trains of definite visual impressions. If a patient in an asylum thinks he is in his own home, and mistakes his attendants for his relatives, he is clearly unable to apprehend that the present environment is different from the environment of his home. This may be due to a failure of the higher apperceptive centres, a real organic incipient dementia, comparable to word-blindness or "mind-blindness" resulting from coarse circumscribed damage of the brain. In the insane a visual disorder of this kind is frequently obscured by other symptoms, and its investigation becomes difficult. In a case of Korsakow's disease, which is described below, Dr. Cole found that the chief stress of the lesion affected the function of vision. The patient was a woman aged 53 years, a labourer's wife. She was a heavy drinker for years and had no children. Except for a "severe chill" in August 1903, she was well until October, when she "suddenly lost her senses, and her legs became useless at the same time." Aural hallucinations followed, she heard "voices" and conversed with imaginary relatives, mistook the nurses for relatives and gave accounts of imaginary journeys. The hallucinations soon subsided, but all the other characteristic symptoms of Korsakow's psychosis were regularly developed. The orientation sense with regard to place was lost, marked amnesia for recent events showed itself, and there was a subjective disorder of vision. She said things looked "misty and blurred," but her visual acuity and colour-sense were normal. Tactile impressions of objects, *e.g.*, a pencil, a penny, a key, paper-knife or purse, were better retained than visual impressions. The results were constant and could not be attributable to caprice. Visual impressions were the most quickly obliterated, and if here and there a visual impression seemed unusually well held, this was due to the auxiliary action of her more retentive auditory memory. She had a very imperfect understanding for pictures. Many common objects of furniture were turned by her in imagination into objects possessed of religious or mystic symbolic significance and influence. Drawing from memory or from copy was equally bad and defective, although she seemed to take great trouble. According to Bonhoefer [3] the defect is, in most cases of Korsakow's malady, greater in relation to hearing than to vision or touch; but he adds that he has seen one case in which visual impressions were effaced with greater facility than auditory, and he mentions cases from Liepmann and Kiefer to the same effect. Dr. Cole's case belongs to the same category. An attempt is made to work out the pathology of this interesting class of cases, but it is recognised that the evidence at present available is not sufficient or conclusive. In Dr. Cole's patient's interpretation of pictures there was also a noteworthy absence of illusions determined by what is known in psychiatry as "flight of ideas" (*ideenflüchtigen Illusionen*). Many of the illusions from which this patient suffered had a "recognisable foundation," says Dr. Cole, and pictures shown to her were fairly well interpreted. It is customary in text-books to distinguish rather sharply two main classes of "mind-blindness," viz., word and letter-blindness, and object-blindness. The recorded mental cases generally presented symptoms of a mixed rather than exclusive type, and though clinico-psychological analysis has not said its last word, "these views," concludes Dr. Cole, "based upon the study of cases of focal lesion, are no less applicable to cases of insanity depending upon diffuse cerebral disorder."

[1] Jour. of Nerv. and Ment. Dis., Nov. 1904. [2] Jour. of Ment. Sci., July 1905. [3] Die akuten Geistenkrankh. etc., Jena 1901, p. 125.

(*To be continued.*)

